# Sequence composition predicts immunoglobulin superfamily members that could share the intrinsically disordered properties of antibody CH1 domains

**DOI:** 10.1038/s41598-017-12616-9

**Published:** 2017-09-29

**Authors:** Max Hebditch, Robin Curtis, Jim Warwicker

**Affiliations:** 1 School of Chemical Engineering and Analytical Science, Manchester Institute of Biotechnology, The University of Manchester, 131 Princess Street, Manchester, M1 7DN UK; 2 School of Chemistry, Manchester Institute of Biotechnology, The University of Manchester, 131 Princess Street, Manchester, M1 7DN UK

## Abstract

Antibodies are central to the growing sector of protein therapeutics, and increasingly they are being manipulated as fragments and combinations. An improved understanding of the properties of antibody domains in isolation would aid in their engineering. We have conducted an analysis of sequence and domain interactions for IgG antibodies and Fab fragments in the structural database. Of sequence-related properties studied, relative lysine to arginine content was found to be higher in CH1 and CL than in variable domains. As earlier work shows that lysine is favoured over arginine in more soluble proteins, this suggests that individual domains may not be optimised for greater solubility, giving scope for fragment engineering. Across other sequence-based features, CH1 is anomalous. A sequence-based scheme predicts CH1 to be folded, although it is known that CH1 folding is linked to IgG assembly and secretion. Calculations indicate that charge interactions in CH1 domains contribute less to folded state stability than in other Fab domains. Expanding to the immunoglobulin superfamily reveals that a subset of non-antibody domains shares sequence composition properties with CH1, leading us to suggest that some of these may also couple folding, assembly and secretion.

## Introduction

Antibodies are key components of the immune system, present in vertebrates from the evolution of jawed fish onwards^1^. As a tool they have proved to be invaluable for clinical^2^ and research purposes^3^, and are the backbone of the burgeoning protein therapeutics (biotherapeutics) portfolio^4^. This latter area in particular is leading to an increased interest in the properties that determine the stability and interactions of antibodies. Compared to other biotherapeutics, antibodies are a favoured affinity platform because of their characteristic high affinity and target specificity, with applications in many different disease sectors^5^.

There can, however, be limitations to the use of therapeutic antibodies in the clinic, where producing suitable pharmaceutical formulations has proved to be difficult^6,7^. A major constraint on biotherapeutics is the high concentration required for storage and delivery in the home-use injection format^8^, facilitating high binding efficacy with wide-spread (non-hospital) use. Antibodies occur at naturally high concentrations in the blood^9^, thus providing a good starting point for stable formulations. The detailed behaviour of antibodies, particularly at high concentrations, is dependent on solution conditions. Research into how sequence and structure influence antibody stability and the formation of soluble and insoluble aggregates is therefore required. Improved understanding in this area would impact on several areas of pharmaceutical drug development, including production, storage and delivery. Identification and engineering of solubility enhancing features in the immunoglobulin framework could improve next generation protein therapeutics. Nevertheless, variations in complementarity determining regions (CDRs)^10^ and framework regions^11^ can lead to significant challenges, for example in minimising aggregation at high concentrations.

CDRs are contained within the variable domains of the heavy and light chains, contributing to fundamental differences in the properties of immunoglobulin (Ig) domains that are formed around the immunoglobulin fold. These differences are of increasing interest as candidate protein therapeutics are constructed from engineered fragments of antibodies. Antibody protein sequence is generally well conserved within a class, except for the CDRs, that determine antigen binding specificity within the variable region of each chain^12^. CDRs are located within the variable Ig domains of the heavy and light chains, with conserved Ig domains forming the framework of the antibody. CH1 domains illustrate the diversity that is possible on a conserved Ig domain fold. The CH1 domain alone is not stable in folded form^13^, requiring proline isomerisation and interaction with the CL domain for folded state stability. This process forms part of the quality control for antibody passage through the secretory pathway^14^. Antibodies must be assembled prior to secretion and function. Where an antibody is not correctly assembled, heavy chains are retained in the endoplasmic reticulum (ER), but isolated light chains can be secreted^15^. Heavy chain retention is due to binding between the CH1 domain and a molecular chaperone (binding immunoglobulin protein, BiP)^15,16^ that recognises the incompletely folded CH1. Addition of light chains can disrupt the heavy chain complex with BiP, leading to assembly and secretion^17^. The importance of CH1 in retaining heavy chains within the ER is demonstrated in studies where CH1 deletion allows the heavy chain to be secreted^18^. Interestingly, despite its intrinsically disordered protein (IDP) nature in isolation, the CH1 domain has been reported to not contain the sequence characteristics of IDPs^14^.

Differences between Ig domain contributions to antibody stability are illustrated by measurements of unfolding rates, in which Fab unfolding is slow compared with that of Fc^19^, with the interaction between CH1 and CL domains in the Fab fragment being maintained even in the presence of sufficient GuHCl to denature individual domains. More recently, it has been suggested that immunoglobulin G (IgG) unfolds in two major steps, the order of which is dependent on the degree to which CDRs destabilise the variable domains^20^. Within the Fc fragment, the CH2 domain has been shown to unfold before the CH3 domain^21^.

Antibody Ig domains are part of the immunoglobulin superfamily (IgSF), a large family of proteins containing Ig domains, with many acting as cell surface receptors^22^. Domains within the IgSF superfamily can be divided according to whether they more closely resemble the variable (V) or constant (C) domain in the immunoglobulin. Many representatives of IgG domains, particularly in Fab fragments are available in the protein structural database (PDB, www.rcsb.org)^23^. Structural analysis of protein domains and their interactions can be accomplished at a simple level through studies of charge interactions and shape complementarity. Methods for predicting charge interactions in proteins have been developed based on continuum electrostatics models. Most ionisable groups in proteins are sited at the water accessible surface, and in this case a Debye-Hückel model with the dielectric value of water is effective^24^. The pH-dependent contribution to folding energy is then calculated from the interactions between ionisable sites using a Monte Carlo scheme for sampling protonation sites^25,26^. These methods give the predicted contribution of ionisable groups to folded state stability of an isolated domain, or of the interaction between domains.

The current work carries out a sequence and structural analysis of IgSF domains, commencing with Fab fragments, as these are abundant in the PDB, studying their variation and confirming that CH1 is an outlier in terms of certain properties. These properties are then analysed for domains in the wider IgSF, which demonstrates a spectrum of similarity to CH1 domains that is orthogonal to sequence identity. It is suggested that a subset of domains within the IgSF may be part of assembly and secretion quality control that is analogous to that mediated for IgG by CH1 domains.

## Results

### Fab domains show distinct sequence-based properties

**Figure 1:**
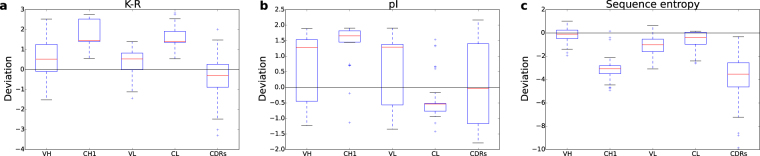
Variation of sequence properties in Fab fragment domains. Various properties are displayed for VH, CH1, VL, CL and CDR regions. CDR sequences were concatenated for each Fab to define one combined CDR sequence for each Fab fragment. Values are plotted as z-score deviations from population averages, using the population standard deviation. Population averages (Niwa) for the *E. coli* proteome^28^ are drawn at the zero deviation line. (**a**) Lysine – arginine composition. (**b**) Isoelectric point, pI. (**c**) Sequence entropy.

Previous work has established that lysine (K) content is, on average, enriched relative to arginine (R) for more soluble proteins^27^. Here, we plot z-score deviation for the property, R percentage composition subtracted from K percentage composition (K-R), relative to the average K-R over the high throughout solubility dataset^28^. Domain median values are higher than the reference value for all domains, with K-R values for constant domains exceeding those of the variable domains in the Fab fragment, and CDRs have the lowest K-R (Fig. 1a). Thus, increased K-R may be associated with higher solubility in mAbs, as appears to be the case for serum albumin, also at high concentration in blood^27^. It is possible that lower values in the variable domains and CDRs affords functional (antigen binding) adaptation, and is balanced with a higher value in the constant domains, reminiscent of engineered solubility fusion tags. It is not clear why arginine may be less favourable than lysine for protein solubility. Discussion has centred on its strength of interaction (compared with lysine) in salt-bridges and cation-pi pairings^27^. Charge interactions generally play a role in antibody stability, and are thought to modulate the resistance of variable domains to aggregation^29^. Overall charge is reflected in the pI (Fig. 1b), with CL clearly different (lower pI, more negative charge) than the other Fab domains. The contrast in pI of CL and CH1 domains indicates that favourable charge interactions could contribute to CL:CH1 dimerisation. CDRs exhibit a broad range of isoelectric points, consistent with variation to match antigen binding requirements.

**Figure 2:**
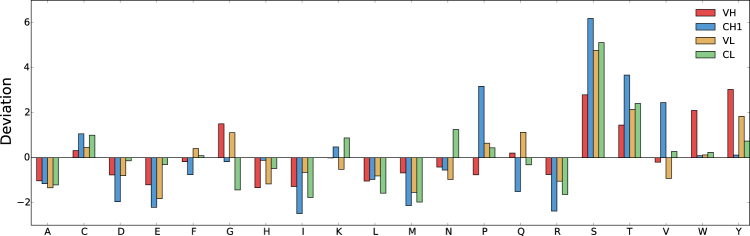
Amino acid compositions of Fab fragment domains. Z-score deviations from population average are shown for the 20 amino acids, for each domain, in the set of Fab fragments.

Sequence entropy is a measure of the degree to which amino acid composition is enriched in some amino acids, with higher values reflecting a more uniform composition across amino acids. This information entropy is calculated from the amino acid compositions of a sequence, and the absolute value ranges between extremes of zero (where the entire sequence is constituted by a single amino acid type) and 4.32 (where a sequence is constituted equally of all 20 amino acids). The CH1 domain and CDRs have relatively low sequence entropy (plotted as deviations from population averages rather than absolute values, Fig. 1c). For CDRs this may indicate enrichment for a subset of amino acids that have more potential for forming interactions with antigens. Studying amino acid compositions (Fig. 2) reveals that CH1 domains, compared with other Fab domains, are enriched for certain amino acids (P, S, T, V), whilst others (D, E, F, I, Q, R, Y) are under-represented. Proline cis-trans isomerisation is known to be involved in the secretion of folded antibodies^30^. Differences in the compositions of other amino acids could give insight to the biophysical features that yield an atypical IDP-like character for the CH1 domain. For example, a smaller number of the bulky and aromatic hydrophobic F and Y for CH1 could indicate that non-polar packing incorporates more smaller hydrophobic groups (e.g. V), with a concomitant increase in the torsional degrees of freedom that require freezing on packing into the folded domain. Non-polar packing would still form the folding core, but with a higher thermodynamic entropy cost for CH1 than for other Fab domains, due to immobilising a greater number of torsional degrees of freedom. Under-representation of charged residues (D, E, R) could give a lower contribution of charge interactions to stabilisation of the folded domain.

**Figure 3:**
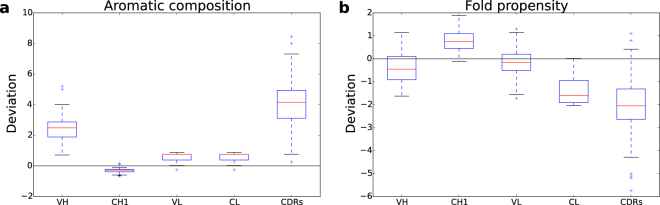
Aromatic amino acid composition and predicted folding propensity of Fab fragment domains. Z-score deviations of properties are shown. (**a**) F, W, Y compositions are combined. (**b**) Predicted folding propensity^32^.

Aromatic amino acid (F, W, Y combined) content was investigated further. Figure 3a emphasises the relatively low aromatic sidechain content of CH1 domains and its high content in CDRs, the latter a well-known property^31^ that presumably results from the key role of CDRs in determining protein-protein interactions. CH1 domains exhibit a restricted range compared with other domains which, along with the lower aromatic content, could be indicative of evolutionary constraint, potentially related to the IDP-like behaviour of CH1 during quality control and antibody export from the cell. An estimate of propensity to form a folded protein can be made based on the balance of hydrophobicity and charge^32^. Surprisingly, this calculation predicts CH1 domains as having the highest folding propensity and least tendency to IDP character, amongst Fab domains (Fig. 3b). The prediction of folding propensity, before scaling to the z-scores shown in Fig. 3b, yields numbers on a scale with >0 predicted as folded. Other than the CDRs, all medians listed in Fig. 3b predict as folded (although CL is close to the folded/unfolded prediction boundary). The hydrophobicity component in the calculation of folding propensity includes all amino acids with non-polar sidechains^32^, so that a low F, W, Y content alone does not necessarily lead to predicted IDP character.

Taken together, these results suggest that, in part, the unconventional IDP-like character of the CH1 domain^14^ can be associated with its sequence composition. Within that composition, the lack of folding stability for CH1 appears to be the result of more subtle effects than the simple balance of hydrophobicity and charge used in a standard measure of folding potential^32^.

### Charge interactions in CH1 are predicted to be relatively small

**Figure 4:**
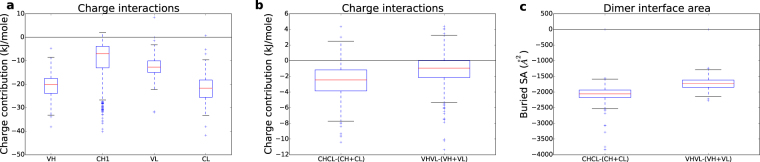
Structure-based properties of Fab fragment domains. (**a**) The predicted contribution of ionisable groups to folding energy for domains. (**b**) Predicted contribution of charged groups to dimerisations within the Fab fragment. (**c**) Molecular surface burial for dimerisations within the Fab fragment.

Interactions between ionisable groups at neutral pH have been predicted for the Fab domains, using continuum electrostatics methods. These are structure-based calculations with results given as absolute values, rather than the deviations to population averages shown for sequence-based calculations. Ionisable groups contribute less to folded state stability for the CH1 domain than the other Fab domains (Fig. 4a). This observation is consistent with the reduced D, E and R sequence composition of CH1, and IDP character for the domain in isolation. It is notable that CH1 has a smaller charge contribution to stability than the variable domains, which might have been expected to sacrifice stability to provide an antigen binding platform. However, the CL domain has greater (predicted) charge contribution to stability than the variable domains, emphasising the difference between CH1 and CL domains. Charge contribution to dimerisation energy is greater for CL:CH1 than for VL:VH (Fig. 4b), but in both cases the contributions are relatively small (averaging −2.5 and −1.0 kJ/mole, respectively). Thus, the large difference in pI of CH1 and CL domains (Fig. 1b) does not translate into a large predicted contribution of charge interactions to dimerisation. Perhaps of more relevance to CL:CH1 dimerisation is a difference in buried surface area, which is greater for CL:CH1 than for VL:VH (Fig. 4c). In summary, CH1 is predicted to possess a lower contribution of ionisable group charge interactions to folded state stability than other domains, and this is not fully compensated with salt-bridging across the CL:CH1 interface. It may be important for CL:CH1 stability (and stabilisation of the CH1 domain fold) that more solvent accessible surface area (SASA) is buried than in the VL:VH dimer.

### Other domains in the immunoglobulin superfamily have features in common with CH1

**Figure 5:**
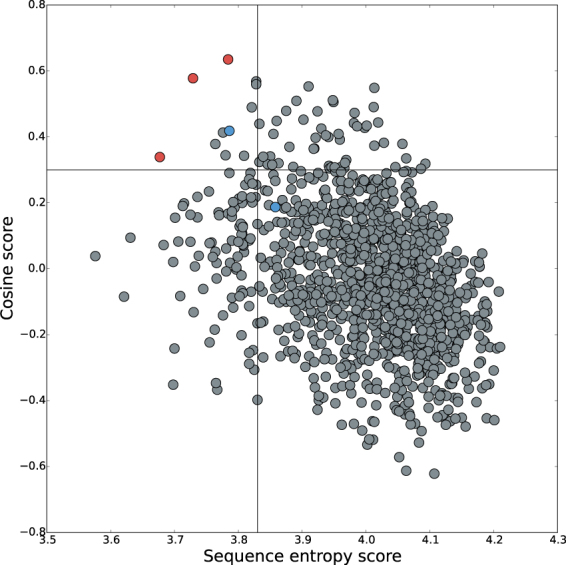
Distribution of human IgSF domains in terms of similarity to CH1 and sequence entropy. Sequence entropy and cosine value for the dot product of vectors (X − CL_REF_) and (CH1_REF_ − CL_REF_) are plotted for the set of human IgSF domains (X). In order to identify domains most similar to CH1 (which has low sequence entropy), a threshold region is indicated for cosine >0.3 and sequence entropy <3.83, which yields 11 domains. The 3 most extreme domains are highlighted (red), and in blue is the only domain of these 11 with a known structure (ICAM3:D1), and a closely related domain (ICAM1:D1).

**Figure 6:**
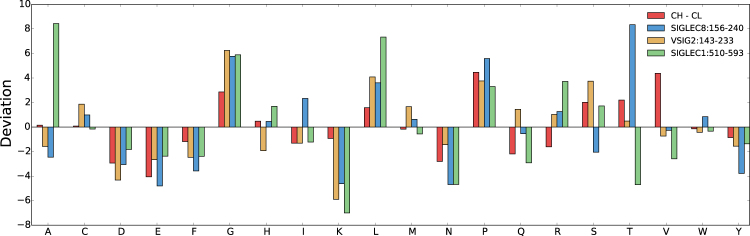
Sequence composition for 3 IgSF domains most similar to CH1 in Fig. 5. Amino acid sequence composition z-score deviations (and, further, as differences to CL) are shown for CH1, and the 3 IgSF domains: VSIG2:142-233, SIGLEC8:156-240, SIGLEC1:510-593.

**Figure 7:**
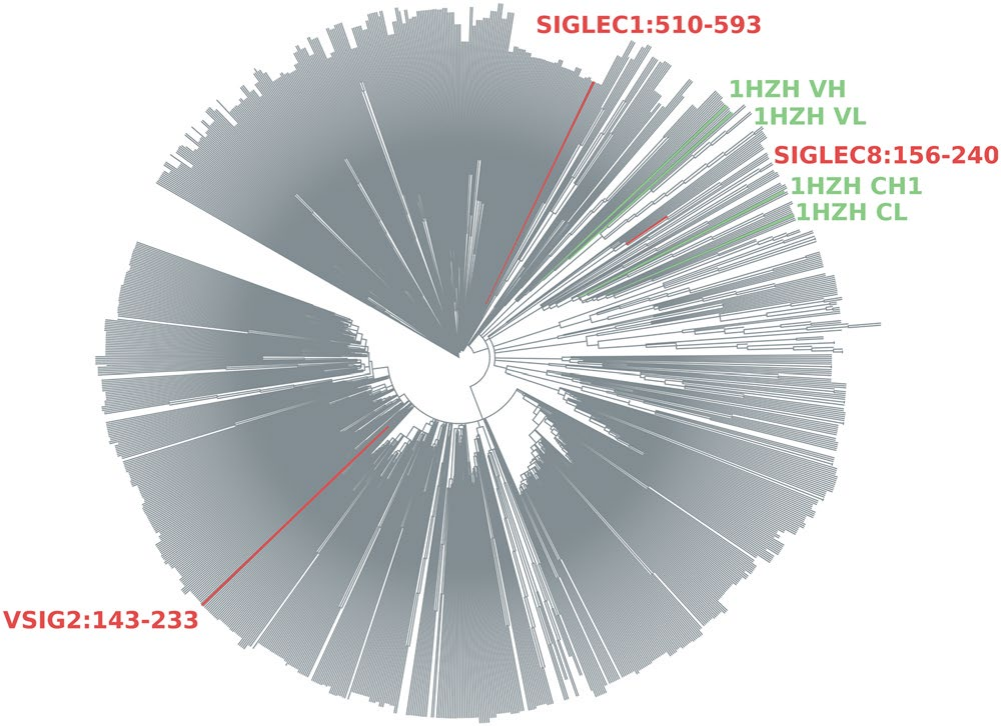
Sequence similarity of selected IgSF domains and CH1. Locations of the 3 IgSF domains of Fig. 6 are shown in this iTOL^48^ plot of similarity from a COBALT^47^ alignment of the human IgSF.

Human representatives of the IgSF^33^ were scanned for similarity of sequence composition to CH1 domains. This method used a vector comparison, based on amino acid compositions, to probe the difference between each IgSF domain and a representative CL domain, against the equivalent difference for representative CH1 and CL domains. The aim of the scan was to emphasise amino composition features (vector components) that differentiate CH1 from CL. As a low sequence entropy also differentiates CH1 from other antibody domains, this property is added to form a double threshold of similarity of IgSF domains to CH1. Figure 5 shows computed IgSF values in this two-dimensional space. Sequence compositions (as deviations from population averages and now also as differences relative to CL) for the three most extreme IgSF domains (VSIG2:143-233, SIGLEC8:156-240, SIGLEC1:510-593, Fig. 5) are shown in Fig. 6. Of the amino acids noted as differentiating CH1 (Fig. 2), depletion of D, E, F, N, and Y is also seen for the three highlighted IgSF domains. In terms of amino acid enrichment in CH1 (Fig. 2), P and G are also enriched in these three domains (Fig. 6). Amino acid compositions for these 3 IgSF domains closely match that of CH1 domains. It is therefore possible that the extent to which composition contributes to the IDP-like properties of CH1 would be replicated in these IgSF domains. Importantly, these similarities are not simply the result of a closer relationship (amino acid identity score) in a multiple sequence alignment. Indeed, these 3 IgSF domains are distributed throughout the IgSF phylogenetic tree (Fig. 7), 2 of the 3 at greater distance from the CH1 domain of crystal structure 1hzh^34^ than is the CL domain from the same IgG.

Having established that some IgSF domains exhibit degrees of similarity to CH1 in terms of sequence-based properties (but not strict sequence similarity), we looked for evidence of 3D domain structures being determined only in the context of domain-domain interactions. Taking thresholds for the cosine similarity parameter (>0.3) and sequence entropy (<3.83, Fig. 5) yields 11 IgSF domains (SIGLEC1:410-507, MDGA2:241-328, SIGLEC1:510-593, HMCN2:1984-2074, SIGLEC7:149-233, ICAM3:45-103, SIGLEC9:145-229, BCAM:362-441, PGBM:2051-2151, VSIG2:143-233, SIGLEC8:156-240), only one of which has an associated structure (domain 1 of ICAM3, 1t0p)^35^. Although ICAM3:D1 can fold independently of other domains^36^, there are a number of interesting features. The closely related ICAM1:D1 (just outside the threshold region in Fig. 5) is reported to form domain-domain interactions upon folding, requiring domain 2 and making D1-D1 homodimer interactions^37^. In comparison, ICAM3:D1 is heavily glycosylated. Alteration of glycosylation in domain 1 of the ICAM family can impede expression at the cell surface^38^. Directed evolution has been used to engineer a stable folding domain variant of ICAM1:D1^39^. Interestingly, mutations at proline and threonine sites were key in establishing the stable folded isolated domain. Thr and Pro feature in the group of amino acids found to be enriched in CH1 domains. Computed ionisable group contributions (pH 7.0) to folding stability for these D1 domains are low at −6.5 kJ/mole for ICAM3 (1t0p, chain B), and −7.8 kJ/mole for ICAM1 (1ic1, chain A), similar to the CH1 domain values (Fig. 4a).

**Figure 8:**
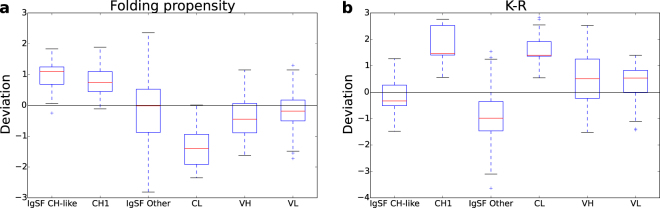
Sequence-based properties of 11 human IgSF domains similar to CH1 in Fig. 5. Comparison between these 11 IgSF domains, other IgSF domains within those 11 proteins, and Fab fragment domains. (**a**) Predicted folding propensity. (**b**) K-R z-score deviation.

Returning to sequence-based properties, calculated for the 11 most CH1-like IgSF domains, folding propensity has a positive deviation from population average (Fig. 8a), as does CH1 (Fig. 3b) and therefore not precluding an IDP-like character for an isolated domain. Interestingly, one sequence feature does differ substantially between the 11 CH1-like IgSF domains and CH1 domains. The K-R property for 11 IgSF domains is lower than that of CH1 (and CL) domains (Fig. 8b). Since these proteins are generally located at the cell surface, rather than being abundant in the cytoplasm, this is consistent with lysine *versus* arginine content constituting an evolutionary constraint for proteins at higher concentration^27^.

## Discussion

Since sequence properties are related to protein solubility^27,28^, we wondered how they would vary between domains within an IgG. Notably, K-R is higher for constant domains than for variable domains. Since lysine is associated with higher protein solubility than arginine, it is possible that the constant domains act in part as solubility tags fused to the variable domains, that must support CDRs adapted for binding affinity rather than solubility. More generally we noted that CH1 domains are outliers in respect of amino acid composition, being enriched for certain amino acids and depleted in others. We wondered whether these properties could be related to the IDP-like nature of the CH1 domain in isolation, and its role in quality control and secretion of mature antibodies^14^. Analysis of charge interactions in 3D structures of Fab fragment domains reveals that CH1 has the lowest predicted ionisable group contribution to folded state stability of a domain, consistent with an intrinsic IDP nature. CH1 domains tend to bury more surface area on complexation with CL domains, than is the case for the VH-VL interface, consistent with the role of CL in stabilising folded CH1. Notably, CH1 domains are not predicted to be IDP-like by a prediction scheme based on the balance of hydrophobic and charged amino acids^32^. With regard to the amino acid composition of CH1 domains, a bias towards non-aromatic sidechains is evident, which would give more torsional degrees of freedom to be immobilised in a folded structure, and thus a destabilisation relative to non-CH1 antibody domains. Variation in amino acid composition between Fab domains leads to sequence entropy being smaller for CH1 domains, signifying a more restricted sampling of amino acid types. We used sequence entropy, together with a measure of amino acid composition deviation from CL, to identify 11 domains in the IgSF that are most similar to CH1. This similarity measure relates to overall amino acid content, rather than sequence identity in a multiple alignment. Considering that these 11 domains may be candidates for the IDP-like properties exhibited by CH1, only one (ICAM3-D1) has a 3D structure reported. Although ICAM3-D1 folds as an independent monomer, it is extensively glycosylated which may increase its folded state stability^35^. A close neighbour, ICAM1-D1 (just below the threshold that selects 11 CH1-like domains), is not extensively glycosylated, and is not stable as an independent domain. We suggest that there may be other domains within the IgSF that possess similar IDP-like character to the CH1domain, and that mediate assembly and secretion in a parallel manner to IgG antibodies. Experience with ICAM1-D1 suggests that such domains could be engineered to acquire folded state stability^39^. Further work could alter CH1 and other IgSF domains to determine key elements (beyond proline content) that determine IDP-like character, and shed light on a set of atypical intrinsically disordered proteins. It will also be of interest to establish the degree to which glycosylation of some IgSF domains contributes to folded state stability. Since protein structural analysis is a convenient indicator of intrinsic stability, this work will advance as the structures of more IgSF domains are mapped.

## Methods

### A dataset of Fab fragment structures

Fab structures were obtained from the PDB using the text search query ‘Fab’ and excluding similarly named structures. Only the heavy (‘H’) and light (‘L’) chain components of structures were retained, missing some occurrences with non-standard naming conventions, but providing a convenient filter for a single copy of the Fab fragment within a larger PDB file. For the dataset of 1119 structures, a degree of heterogeneity in chain length was observed. In order to avoid scanning of all structures individually, a further filter was employed, based on heavy and light chain lengths. Lower and upper quartiles of chain length were derived for both chains, and structures with chain lengths between these values selected (between 219 and 228 amino acids for heavy chain fragments and between 213 and 218 amino acids for the light chain). This reduced the dataset to 387 Fab structures.

The interdomain regions between the constant and variable domains of heavy chain fragment and light chain were identified by studying a small number of the models visually using the molecular graphics software Visual Molecular Dynamics (VMD)^40^. Interdomain regions were found between residues 110 and 130 for the heavy chain fragment, and residues 100 and 115 of the light chain. In order to determine break points between domains more precisely, the amino acid sequences of heavy chain fragments and light chains were aligned using MUSCLE^41^, and the interdomain regions searched for conserved sequence interdomain motifs. Of the 387 heavy chain fragment sequences, 375 were found to contain a *VS* motif between residues 110 and 130, followed by either an *S* or *A*. For the light chain, two search motifs were used. The majority followed a *E*[*LIV*]*KR* pattern (311/387). To increase this number, the search pattern *TVL*[*GSA*] was also identified, present in 44 light chain interdomain regions. One or other of these motifs could be found in 355 of the 387 light chain interdomain regions between residues 100 and 115.

Combining motif filtering for heavy and light chains gave 350 Fab structures from 387, for which domains were obtained from splitting at the interdomain motifs. A further filter was applied, requiring that all domains had a length within 10 amino acids for the mean for that domain in the 350 subset, with a decrease to 333 Fab fragments and associated domains (VL, CL, VH, CH1), available as structures and sequences. Structures were also extracted for VL:VH and CL:CH1 domain dimers. The dataset used for calculations therefore consisted of 333 Fabs, 666 VL:VH/CL:CH1 dimers, and 1332 individual immunoglobulin domains. Using the SAbDab database^42^, the CDR regions for 247 of the Fabs were identified, and the resulting amino acid sequences formed a CDR dataset.

### Sequence and structure-based calculations

Sequences were processed using software developed in our group that calculates amino acid compositions and other properties, such as pI, sequence entropy (a measure of the extent to which a protein is enriched in a subset of amino acids), and folding propensity (based on a balance between hydrophobicity and charge)^32^. Additionally, lysine and arginine composition are compared, as previous work indicates that lysine may be preferred over arginine in more soluble proteins^27^. In analysis of sequence-based properties, calculated values are given as deviations from population averages in a high-throughput dataset of *Escherichia coli* protein solubility (derived from quantification of protein expressed in soluble and insoluble forms)^28^. This allows comparison both between the subsets being studied (e.g. CH1 and other IgG domains), and of these subsets to a proteome average, albeit the *E. coli* proteome. The deviation value is the z-score measure of how many population standard deviations a property is away from the population average (positive or negative).

Structures were investigated using continuum electrostatics for predicting the contribution of ionisable groups to structural stability^43^. A Debye-Hückel model^24^, was used to generate the pH-dependent component of folding energy. This method uses a partitioning of ionisable group energy^25^, together with Monte Carlo sampling of ionisation states^26^, to predict pKas and pH-dependent energy. The value of ionisable group interactions at pH 7.0 was then extracted, as an estimate of the contribution of ionisable group interactions to folded state stability. Calculation of SASA was made with in-house code, partitioning according to contribution by polar or non-polar atoms. Contributions to dimerisation (VL:VH, CL:CH1) were calculated, either for electrostatic energy or SASA, by subtraction of the individual subunit contributions from that of the dimer.

IgSF domain sequences were aligned with clustal^44^, and Biopython^45^ used in the processing.

### The immunoglobulin superfamily

To make wider comparisons, identifiers for other members of the immunoglobulin superfamily (IgSF) in humans were obtained^33^, and corresponding sequences retrieved from UniProt^46^. The 477 identifiers were cross-referenced with UniProt and 1229 immunoglobulin domain amino acid sequences extracted. The human IgSF sequence dataset was also processed to give amino acid compositions. To compare amino acid composition between CH1 and other IgSF domains, code was written to create a 20-dimensional vector of amino acid composition values. A representative CL domain composition (CL_REF_) was subtracted from a representative CH1 domain (CH1_REF_) and IgSF domain composition vectors, to increase sensitivity to the differences between CH1 and CL domains. For an IgSF domain X, the vector dot product, (X − CL_REF_). (CH1_REF_ − CL_REF_), was calculated and used to derive the cosine of the angle between these differenced vectors. A cosine close to zero relates to little similarity, whilst close to 1 is highly similar i.e. the closer the cosine to 1, the more similar is the deviation of domain X from CL_REF_ to the deviation of CH1_REF_ from CL_REF_. In this way, it is possible to assess the degree to which an IgSF domain recapitulates the features that separate CH1 from CL domains. This measure of similarity was combined with sequence entropy to visualise in a 2D plot those IgSF domains most similar to CH1. Visualisation of sequence alignment for the entire human IgSF using COBALT^47^ was made with iTOL^48^.

### Data Availability

The datasets analysed during this study are available from the corresponding author on reasonable request.
